# Perception of recurrence risk in patients with IgG4-related disease: a descriptive phenomenological study

**DOI:** 10.1093/rap/rkae148

**Published:** 2024-12-04

**Authors:** Kai Yu, Hui Gao, Shibo Liu, Haihong Yao, Xiaoqing Wang, Chenghua Luo, Qian Guo, Yuexian Shi

**Affiliations:** School of Nursing, Peking University, Beijing, China; Department of Rheumatology and Immunology, Peking University International Hospital, Beijing, China; Department of Retroperitoneal Tumor Surgery, Peking University International Hospital, Beijing, China; Department of Rheumatology and Immunology, Peking University People’s Hospital, Beijing, China; School of Nursing, Peking University, Beijing, China; Department of Retroperitoneal Tumor Surgery, Peking University International Hospital, Beijing, China; Department of Rheumatology and Immunology, Peking University International Hospital, Beijing, China; School of Nursing, Peking University, Beijing, China

**Keywords:** IgG4-related disease, recurrence, perception, qualitative

## Abstract

**Objective:**

About 40% of IgG4-related disease (IgG4-RD) patients face recurrence, severely impacting their quality of life. We aimed to explore the characteristics of the perception of recurrence risk in patients with IgG4-RD.

**Methods:**

A qualitative study design with a descriptive phenomenological approach was used. Fourteen patients with IgG4-RD were recruited via purposive sampling, including six patients with first onset and eight patients experiencing recurrence. Semi-structured interviews were conducted to collect data, and transcripts were analysed by two independent researchers using Colaizzi’s descriptive analysis framework. The COREQ checklist was followed.

**Results:**

Data analysis identified nine subthemes falling into four themes: (a) perception of differential susceptibility to recurrence; (b) perception of crucial recurrence risk factors; (c) perception of recurrence warning signs and medical behaviours; (d) perception of multiple recurrence outcomes. We found that susceptibility perception formed the basis of recurrence risk perception. For patients with first onset, the main manifestations were misconception or evasion of the risk of recurrence, whereas patients experienced recurrence demonstrated a clear perception of recurrence risk and feelings of fear. Based on this, other themes emerged.

**Conclusions:**

Since the absence of accurate knowledge related to recurrence, the perception of recurrence risk in patients with IgG4-RD primarily manifested as misconception, evasion or feeling fear. Ultimately, they couldn’t take appropriate actions to prevent recurrence. Healthcare professionals should develop comprehensive interventions for patients with IgG4-RD, integrating health education, disease consultation and psychological support, with the aim of enhancing awareness of recurrence risk and empowering them to manage their conditions in the long term.

Key messages

- The perception of recurrence risk in IgG4-RD patients includes four main aspects.
- Patients’perceptions of recurrence risk often manifest as misunderstanding and fear.
- IgG4-RD patients lack timely access to knowledge to prevent and address recurrence effectively.

## Introduction 

IgG4-related disease (IgG4-RD) is a newly recognized chronic fibro-inflammatory disease of unknown aetiology [1]. Clinical manifestations of IgG4-RD include autoimmune pancreatitis, IgG4-related ophthalmic disease, retroperitoneal fibrosis, autoimmune cholangitis, interstitial pneumonia, etc. [2]. Previous studies reported that the recurrence rate of IgG4-RD after induced remission therapy was approximately 40% [3, 4]. Recurrence can lead to poor prognosis [5] and, as a result, to increase readmission rates and rehabilitation costs, placing a substantial burden on the patients, families, and society [6]. Therefore, it is crucial to prevent recurrence for the health management of patients with IgG4-RD.

We performed a comprehensive literature review and found that IgG4-RD recurrence research had primarily focused on risk factors, which could be classified into three main categories, including pathophysiological factors, genetic and familial factors, and unhealthy lifestyle and behavioural factors [7–9]. Among these factors, the impact of unhealthy behaviours and lifestyle on recurrence is particularly prominent and is the only factor that can be modified and controlled [10]. According to the Health Belief Model [11, 12], patients are more likely to adopt positive coping measures for prevention when they can accurately perceive the risk and severity of recurrence, thereby reducing the risk of recurrence.

The definition of perceived risk of recurrence was derived from risk perception (possibility and consequences) and disease perception (actual situation and influencing factors) [13, 14]. To the best of our knowledge, studies on perception of recurrence risks have mainly focused on cancer and cerebrovascular diseases. Rotgerink found that over one-third of breast cancer patients underestimate their risk of recurrence [15]. Similarly, Lin *et al.* discovered that stroke survivors, even those who have experienced multiple incidents, often lack a clear understanding of their risk of recurrence [16]. This reveals a significant gap in timely, accurate, and systematic access to information related to recurrence. A lack of awareness often leads to neglect and underestimation of the risk of disease recurrence. Without a clear understanding of their relapse risk, patients are unable to take appropriate preventive measures. Since the pathological mechanisms and therapeutic approaches of IgG4-RD differ markedly from other common chronic diseases, the perception of recurrence risk in patients with IgG4-RD would be different, which impacts whether timely and standardized treatments will be received. Currently, there is a lack of studies on the perception of recurrence risk of IgG4-RD. Qualitative methodology allows researchers to observe and understand behaviours and attitudes from the perspective of patients [17]. Here, we adopt a qualitative research method to define the main content of perception of recurrence risk in IgG4-RD patients. Through in-depth exploration and description, we provide new insights to support the treatments of IgG4-RD.

## Methods

### Study design

A descriptive phenomenological study was conducted on the basis of Colaizzi’s study (1978), which provided a phenomenological approach to understanding the perception of recurrence risk in IgG4-RD patients by exploring their perceptions and feelings. This study was designed and reported according to the consolidated criteria for reporting qualitative studies (COREQ) checklist [18].

### Sampling and recruitment of participants

The study was conducted in Beijing, China, from September 2023 to February 2024. We used purposive sampling to select participants through the inpatient and outpatient services of a hospital. The inclusion and exclusion criteria for these participants are depicted in Table 1. Retrieval of no new information from two consecutive interviews was used to determine data saturation and discontinuation of the interviews. A total of 14 IgG4-RD patients were recruited, and all of them completed the study.

**Table 1.** Inclusion and exclusion criteria of participants

Inclusion criteria: 1. Age ≥ 18 years old; 2. Meeting the comprehensive diagnostic criteria for IgG4-related disease [19]; 3. Clear consciousness and willingness to participate in the study; 4. Signed informed consent.
Exclusion criteria: 1. Patients who diagnosed with mental illness, such as schizophrenia, depression, dementia, and epilepsy.

### Data collection

The interview settings were independent, quiet, and private, where participants could express their experiences freely. Before conducting semi-structured interviews, the researchers received training and mastered the skills to perform qualitative research. According to the aim of the study, the interview outline (Table 2) was initially formulated and revised by consulting experts. Prior to the interviews, the participants were informed of the study’s purpose and process, as well as the principle of privacy. Furthermore, they were required to complete an informed consent form. Their interviews, lasting for 40–60 min, were recorded. A few techniques were employed to improve the interviews, which included breaking the deadlocks, building trust in relations through verbal and non-verbal words, allowing adequate time for reflection and response, and ensuring that the participants understood the questions by repeating them. The interviews were conducted in Chinese and translated into English.

**Table 2.** Outline of the interview

1. What is your current understanding of disease recurrence? How did you acquire this knowledge? 2. What factors do you believe may contribute to disease recurrence? Please elaborate on your reasons. 3. What signs may precede disease recurrence? How do you determine this? 4. What impact disease recurrence may have on you? 5. What have your experiences been like since you learned of your recurrence? (Ask this question to patients with recurrence) 6. What measures have you already taken or planned to take to prevent recurrence? Why? What are their (anticipated) effects?

### Data analysis

We used NVivo 11 to code and manage all interview data. The data transcribed by two researchers were analysed in seven steps by utilizing the qualitative data analysis method [20]. The steps were as follows: (1) researchers read the transcribed texts repeatedly to understand the information conveyed in the interviews; (2) researchers re-read each text critically and identified significant statements related to the subject of the study; (3) meaningful sentences and phrases were extracted as initial codes; (4) through constant comparison, repeated codes and categories were aggregated into themes; (5) themes were linked closely to research phenomena and detailed; (6) essential structure constituting the phenomenon was stated; (7) the results were fed back to the participants to confirm the authenticity of the content.

### Bracketing, reflexivity, and rigour

To reduce subjective factors and improve the research quality, we controlled the following factors during the interview:

- Given the sensitivity of the topic, we established relationship with participants by listening and using a non-judgemental approach. We ensured that the interview questions were neutral and open-ended to encourage participants to share their opinions and experiences.
- To describe the experiences of the participants from a research perspective as impartially as possible, we bracketed the preconceptions of the phenomenon by adopting a multidisciplinary team approach to interviewing and data analysis. The two authors (K.Y. and X.W.) analysed the data, and differences in interpretations were resolved by discussion. Therefore, the risk of imposing personal views on the analysis was reduced.
- The credibility and originality of the analysis were obtained by gathering rich, in-depth data from interviews and transcribing verbatim and the participants’ own words, ensuring that our interpretations of the data were drawn from and evidenced by paradigm extracts from the interview data.

### Ethical approval and informed consent

This study was approved by the Ethics Committee of Peking University International Hospital (2023-KY-0035-02). Before the interviews, all participants were informed about the study, their rights, possible discomfort, and expected benefits, while they signed an informed consent. To respect the wishes and privacy of the participants, the length of the interviews could be decided by them, and their names were coded.

## Results

A total of 14 patients with IgG4-RD were interviewed in this study, including 6 of first onset and 8 with recurrences. Other information is shown in Table 3.

**Table 3.** Sociodemographics of patients with IgG4-RD

Characteristics	IgG4-RD (*N* = 14)
Age range (years old)	26–80
Educational level	
Elementary	3
Lower intermediate	5
Upper intermediate	6
Family monthly income	
<￥3000	5
￥3000–6999	8
≥￥7000	1
Marital status	
Single	1
Married	11
Divorce	2
Episode	
First onset	6
Recurrences	8

**Figure 1.**
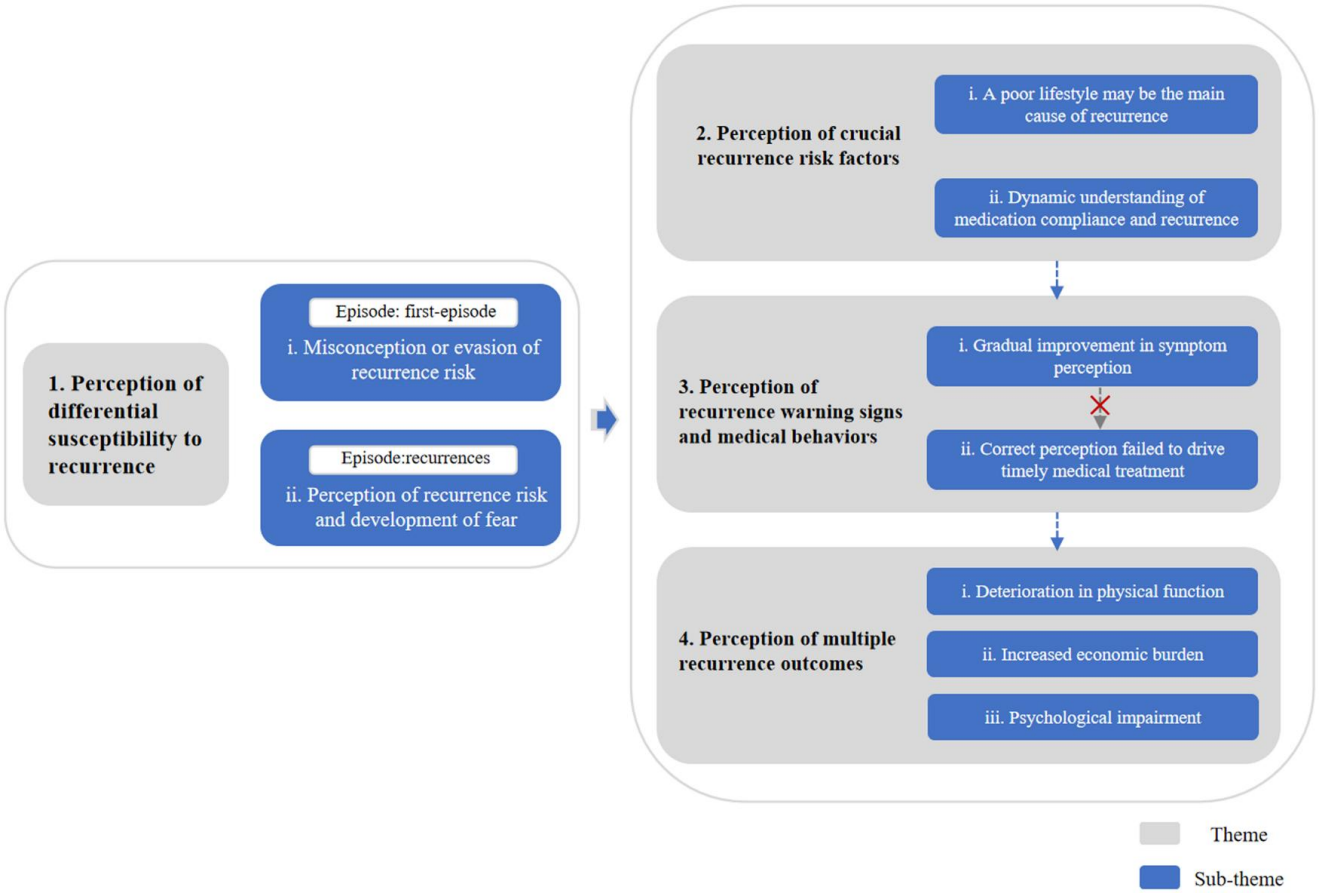
Frame diagram of perception of recurrence risk in patients with IgG4-RD

We identified nine subthemes that fell into four themes (see Table 4). We found that susceptibility perception formed the basis of recurrence risk perception. For patients with first onset, the main manifestations were misconception or evasion of the risk of recurrence, while patients who had experienced recurrence demonstrated a clear perception of recurrence risk and feelings of fear. Based on this, three themes emerged: perception of crucial recurrence risk factors, perception of recurrence warning signs and medical behaviours, and perception of multiple recurrence outcomes. There were a series of problems both in the perception of recurrence risk factors and warning signs in patients with IgG4-RD. On the one hand, patients with IgG4-RD lacked disease-specific knowledge as well as access to it. On the other hand, patients with IgG4-RD gradually enhanced their ability of symptom perception, but correct perception failed to drive timely medical treatments. These have resulted in a set of serious outcomes (see Fig. 1).

**Table 4.** Themes and subthemes

Themes	Subthemes
*Theme 1*: Perception of differential susceptibility to recurrence	*Subtheme 1*: Misconception or evasion of recurrence risk
	*Subtheme 2*: Perception of recurrence risk and development of fear
*Theme 2*: Perception of crucial recurrence risk factors	*Subtheme 1*: A poor lifestyle may be the main cause of recurrence
	*Subtheme 2*: Dynamic understanding of medication compliance and recurrence
*Theme 3*: Perception of recurrence warning signs and medical behaviours	*Subtheme 1*: Gradual improvement in symptom perception
	*Subtheme 2*: Correct perception failed to drive timely medical treatment
*Theme 4*: Perception of multiple recurrence outcomes	*Subtheme 1*: Deterioration in physical function
	*Subtheme 2*: Increased economic burden
	*Subtheme 3*: Psychological impairment

### Perception of differential susceptibility to recurrence

Perception of recurrence susceptibility refers to the subjective assessment of the likelihood of IgG4-RD patients experiencing a recurrence. Our study revealed notable disparities in the perception of susceptibility to recurrence between patients who experienced their first onset and recurrence. The former primarily displayed misconceptions or evasion of the susceptibility of recurrence, while the latter demonstrated an enhanced ability to perceive the susceptibility of recurrence and with consequent concerns and fear.

### Misconception or evasion of recurrence risk

Most participants who experienced first onset had misconceptions about the recurrence risk, equating symptom improvement with disease recovery and feelings that they would not experience recurrence, particularly in the absence of any sequelae after timely diagnosis and treatment.

P2: If it’s cured, then there shouldn’t be any problem, right? I don’t think it will recur.

Furthermore, patients with first onset were unwilling to associate with the recurrence even after they perceived the possibility of disease recurrence, manifesting as evading the topic of recurrence risk.

P4: I never thought it would recur. Just need to have regular check-ups, don’t think about it, it’s not auspicious to always think about it.

### Perception of recurrence risk and development of fear

For patients with recurrence, they generally had a more accurate understanding of the objective fact that there had always been a risk of recurrence.

P1: Although there aren’t many patients with this disease, the risk of recurrence is quite high. I don’t understand this until after my own recurrence.

However, due to the exacerbation of symptoms and the poor prognosis after recurrence, some participants expressed fears of recurrence risk.

P5: I know the risk of recurrence for this disease is quite high. Now, whenever someone mentions recurrence, I get scared.

### Perception of crucial recurrence risk factors

The perception of recurrence risk factors refers to the recognition and understanding of the factors that may lead to recurrence of IgG4-RD. In this interview, poor lifestyle and medication compliance were important factors frequently mentioned by participants.

### A poor lifestyle may be the main cause of recurrence

Patients with IgG4-RD who experienced both first onset and recurrence mentioned that they had poor lifestyle such as staying up late, smoking, or excessive drinking. They felt that these behaviours reduced physical fitness and made disease easier to recur.

P3: I couldn’t sleep at night, my sleep was disturbed, and I suspect that staying up late had something to do with recurrence.

P6: I don’t have any hobbies, except smoking. (I used to smoke) about 2 boxes a day, which may lead to recurrence.

Participants expressed that the information about recurrence risk factors did not come from authoritative medical advice, but from non-professional sources such as conjecture, empirical inference, or searching internet.

P3: I never talked about factors that may lead to recurrence with the doctor, this is all my own thinking after being ill, sometimes I also search Baidu (a Chinese language-based search engine).

### Dynamic understanding of medication compliance and recurrence

Due to the severity of symptoms and the therapeutic effects of medication, patients with first onset recognized the importance of adhering to medication and having strong compliance.

P2: Taking glucocorticosteroid is particularly effective for this disease, and the doctor said that we must adhere to the medicine.

However, patients with recurrence expressed that as their symptoms improved and medication side effects appeared, they would attenuate or discontinue medication. Some patients with first onset also believed that maintenance treatment was no longer necessary once the symptoms subside.

P7: This medicine has side effects, including hyperglycemia. Once the disease has been cured, I will stop this medicine.

Unfortunately, recurrence occurred when medication was attenuated or discontinued, forcing patients to initiate a new stage of treatment at the cost of increasing dosage. Consequently, patients find themselves trapped in a vicious cycle of symptom improvement, irregular medication usage, and disease recurrence. After experiencing multiple recurrences, patients gradually realized the importance of regular medication intake, and then their medication adherence improved.

P5: Last time, I was fat because of glucocorticosteroid. Then, I decided to take two less pills a day, and gradually stopped. Finally, my disease returned. I will definitely follow the doctor’s advice in the future.

### Perception of recurrence warning signs and medical behaviours

The perception of recurrence warning signs refers to IgG4-RD patients using their knowledge or experiences of the disease to identify early symptoms of recurrence. In this study, patients with first onset could not perceive warning signs of recurrence. But with accumulated disease experience and an increased number of recurrences, their ability to perceive warning signs of recurrence gradually improved. However, after perceiving warning signs, some patients still did not actively seek medical attention, ultimately leading to delayed treatment.

### Gradual improvement in symptom perception

Almost all patients with first onset did not know about the warning signs of recurrence.

P4: I really don’t understand this. No one told me before. What kind of symptoms indicate recurrence?

After experiencing recurrences, patients usually gained a preliminary understanding of the warning signs of recurrence. Some patients expressed that their experiences of recurrence had, to a certain extent, heightened their sensitivity to the warning signs of recurrence.

P5: Last time, the right side of my abdomen ached when I had a recurrence. So, when I had that symptom again, I knew it may recur.

### Correct perception failed to drive timely medical treatment

Although some patients accurately recognized the signs of recurrence, they were unwilling to seek medical treatment. They believed that if the symptoms did not significantly affect their daily life, there was no need to seek medical attention.

P3: I think I’m quite tough. It (the symptoms) will pass after a while, and it doesn’t affect me much. So, there is no need to go to the hospital.

In addition, patients often attempted to alleviate symptoms by adjusting their medication themselves. However, the results were usually unsatisfactory, leading to treatment delays.

P7: When the symptoms reappeared, I started to take an extra half tablet of my medication every day. But unfortunately, I couldn’t control the symptoms afterwards, so I came to the hospital.

### Perception of multiple recurrence outcomes

The perception of recurrence outcome refers to the conjecture regarding the outcomes of recurrence and the actual perception after experiencing recurrence, which included deteriorated physical function, additional financial burden, and impaired psychological status.

### Deterioration in physical function

Patients with IgG4-RD indicated that the deterioration of physical function was one of the most prominent consequences of recurrence, including fatigue, decline of immune system function, and overall weakness.

P4: If there’s a recurrence, it will definitely affect my body. It won’t recover to the state before getting sick.

Moreover, compared with patients with first onset, those who have experienced recurrence further recognized that high-dose steroid could exacerbate the deterioration of physical function, such as osteoporosis or hyperglycaemia.

P1: After the recurrence, I will receive a high-dose steroid treatment again. Taking this steroid now, I feel weak all over, walking feels heavy, and develop hyperglycemia.

### Increased economic burden

Patients with multiple recurrences incurred significant financial expenses during disease treatments, leading to their concerns about increased family economic burden.

P3: I have to spend more money every time I recurrenced, and I can’t work at all. It’s also a considerable economic burden for my family.

The economic burden on patients with recurrence arose from various factors, including expenses for out-of-town treatments, travel expenses for follow-up, medication costs, examination fees, as well as economic shortages due to unemployment or work stoppages.

P5: I’ve been running to the hospital all year. That cost me a lot of money. I don’t have a pension, and it’s really becoming a burden.

### Psychological impairment

On the one hand, multiple recurrences have caused patients to constantly suffer from the pain and torment of the disease, severely affecting their quality of life. The fear of recurrence led to a series of psychological impairment, especially anxiety.

P7: When it (IgG4-RD) recurs, I am worried that my body may deteriorate as a result. I just want to live a healthy life for a few more years.

On the other hand, since IgG4-RD could not be completely cured, the uncertainty of when the disease will recurrence and how severe it might be further led patients to feel helpless and hopeless.

P1: This disease often relapses. I’ve been to the hospital so many times. I feel like I won’t fully recover in my lifetime. I really don’t know how to get rid of this disease.

## Discussion

In this study, it was evident that patients with IgG4-RD perceived susceptibility to recurrence differently. The first onset displayed misconceptions or evasion of the risk of recurrence. The main reason was that most patients have limited knowledge of IgG4-RD due to its rarity and complexity. Additionally, those with limited financial resources often faced challenges in accessing sufficient information and support services, which exacerbated their misconceptions about recurrence risk. Furthermore, it might be influenced by traditional Chinese culture, as patients held the belief that discussing negative outcomes might make them come true [21] and thus were reluctant to discuss or confront recurrence. In contrast, patients who had experienced recurrence were more likely to recognize that the risk of IgG4-RD recurrence was high, leading to a psychological fear and worrisome about the progression of disease. Based on the Cognitive Information Processing Theory [22], patients could develop diverse perceptions and take actions based on how they process information. It further confirmed the instability and individual differences in the susceptibility perception of recurrence of patients with IgG4-RD. Given the heterogeneity in perception of recurrence susceptibility of patients with IgG4-RD, it is advised that medical professionals conduct timely assessments and explore more targeted interventions.

Our study found that patients with IgG4-RD had a limited recognition of the risk factors for recurrence. The majority of participants lacked not only disease-specific knowledge and recurrence risk awareness, but also access to authoritative disease information. Even worse, there were many uncertainties among the information from the experience of themselves and non-medical platforms. Pauer *et al.* showed that information from medical institutions is most reliable [23]. Therefore, it is necessary to explore channels that medical staff can provide systematic and accurate information to patients with IgG4-RD anytime and anywhere. The digital health management model based on the Internet and mobile platform [24, 25] has been widely recognized and could be considered. The study also found that medication adherence of patients with IgG4-RD fluctuated depending on the symptom severity and medication side effects. Expert consensus emphasized that it was important for patients with IgG4-RD to take medicine regularly to prevent recurrence [26]. In order to improve medication adherence, healthcare professionals should encourage patients’ involvement in decision-making by allowing them to express their preference and opinion in treatment.

This study found that patients with IgG4-RD were unable to identify and respond to the warning symptoms timely. On the one hand, most first-onset patients showed a limited ability or lack of confidence in identifying recurrence warning symptoms. This could be explained by the following reasons. As a rare disease, there is an insufficient education of IgG4-RD, leading to a lack of awareness about its symptoms and prognosis among general populations [27]. On the other hand, it was notable that patients with IgG4-RD may still delay the specialized treatment and shorten their course of therapy even after experiencing real recurrence process. This could be attributed to the cost of medical treatments. At present, only a few hospitals have the ability to diagnose and treat IgG4-RD [28, 29], and these hospitals are mainly located in first-tier cities such as Beijing in China. Therefore, for patients outside first-tier cities, there are direct (travel, accommodation, and time) and indirect financial burdens (lost income) associated with going to designated hospitals in the cities and returning back to their hometown irregularly [30]. In this case, the neglect for symptoms from patients with IgG4-RD led to delay treatment. This necessitates measures to increase caution of patients with IgG4, enabling them to play positive roles in identifying warning symptoms of recurrence and receiving timely treatments. Furthermore, emerging technologies such as telehealth may additionally facilitate early access to specialized treatment for patients with IgG4-RD when regular providers were not available by enabling electronic consultations (e-consults) and remote monitoring services [31].

In this study, patients with IgG4-RD perception of recurrence outcomes mainly manifested in physiological, psychological, and economic aspects. From a physiological perspective, IgG4-RD recurrence was typically accompanied by exacerbation of symptoms, which led to a decline in physical functions. Similar results were obtained by Zongfei *et al.*’s study [5]. Economically, recurrence implied prolonged disease progression, which not only required additional medical expenses but also forced patients to take time off work, resulting in a loss of income for a period of time. Gong *et al.*’s research pointed out that rare diseases were often excluded from the medical insurance system [32]. To address the issue, coordination of social resources, encouragement of donations, and establishment of a model where costs were shared among medical insurance funds, individuals, and third-party sponsors should be considered based on successful policies from other developed countries. Finally, the participants in this study expressed the concerns that recurrence could lead to increased psychological burdens, such as anxiety, depression, and so on. Therefore, we recommend that the treatments and management of IgG4-RD patients should incorporate mental health care into their treatment plans. This includes using screening tools to assess psychological issues such as the Hospital Anxiety and Depression Scale (HADS), which evaluates anxiety and depression levels. For high-risk IgG4-RD patients, implementing strategies such as mindfulness programmes, psychological counselling, and support groups can significantly enhance their overall well-being and quality of life.

Our study has several limitations. The participants were recruited from a single hospital in China, thus the results might not be generalized before being confirmed by multicentre research. Besides, based on our study, longitudinal qualitative research should be considered to obtain a more comprehensive and in-depth understanding of the experience of individuals or groups over time. Additionally, including longitudinal quantitative research is essential for effectively analysing the trajectory of the influencing factors related to different perceptions of recurrence risk.

In conclusion, the risk perception of recurrence among patients with IgG4-RD mainly included susceptibility perception, risk factor perception, warning symptom perception, and outcome perception. Overall, patients with IgG4-RD generally had a limited perception of the risk of recurrence. They were unable to timely, accurately, and systematically obtain knowledge related to recurrence. In the absence of an accurate perception of the risk of recurrence, patients could not take appropriate actions to prevent or deal with recurrence. Therefore, it is strongly recommended that healthcare professionals need to develop comprehensive interventions for patients with IgG4-RD, integrating health education, disease consultation, continuous monitoring, and psychological support, with the aim of enhancing awareness of recurrence risk and empowering them to manage their conditions in the long term.

## Data Availability

Data are available upon reasonable request.
